# Supplementary material for: The hiuABC Operon Mediates Xenosiderophore Utilization in Caulobacter crescentus
Source: bioRxiv. 2025 Sep 4:2025.09.04.674318. Preprint. [Version 1] doi: 10.1101/2025.09.04.674318 (PMC12424826; doi:10.1101/2025.09.04.674318)
Supplement: 1 [file NIHPP2025.09.04.674318V1-supplement-1.pdf]

bioRxiv preprint doi: https://doi.org/10.1101/2025.09.04.674318; this version posted September 4, 2025. The copyright holder for this preprint (which was not certified by peer review) is the author/funder, who has granted bioRxiv a license to display the preprint in perpetuity. It is made available under aCC-BY 4.0 International license.

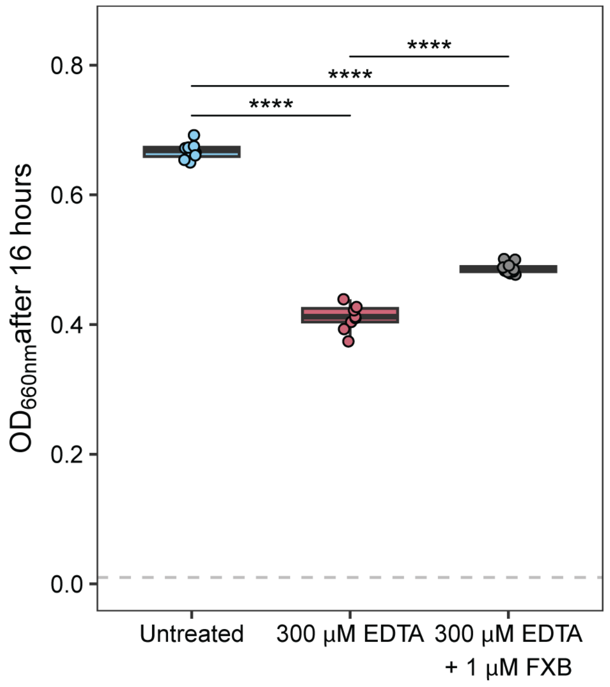

Figure S1. Ferrioxamine B enhances the growth yield of *C. crescentus* in iron-limited PYE broth.
Optical density (OD_{660}) of strains after 16 hours of growth in PYE broth under the following conditions: untreated (blue), supplemented with 300 µM EDTA (pink), or with 300 µM EDTA and 1 µM ferrioxamine B (FXB). Box plots show the median and interquartile range (25th and 75th percentiles), overlaid with individual data points for each independent culture (n = 9). Statistical comparisons of OD_{660} values were performed using a Kruskal–Wallis test followed by Dunn's post hoc test. Significance: ****P < 0.0001.

bioRxiv preprint doi: https://doi.org/10.1101/2025.09.04.674318; this version posted September 4, 2025. The copyright holder for this preprint (which was not certified by peer review) is the author/funder, who has granted bioRxiv a license to display the preprint in perpetuity. It is made available under aCC-BY 4.0 International license.

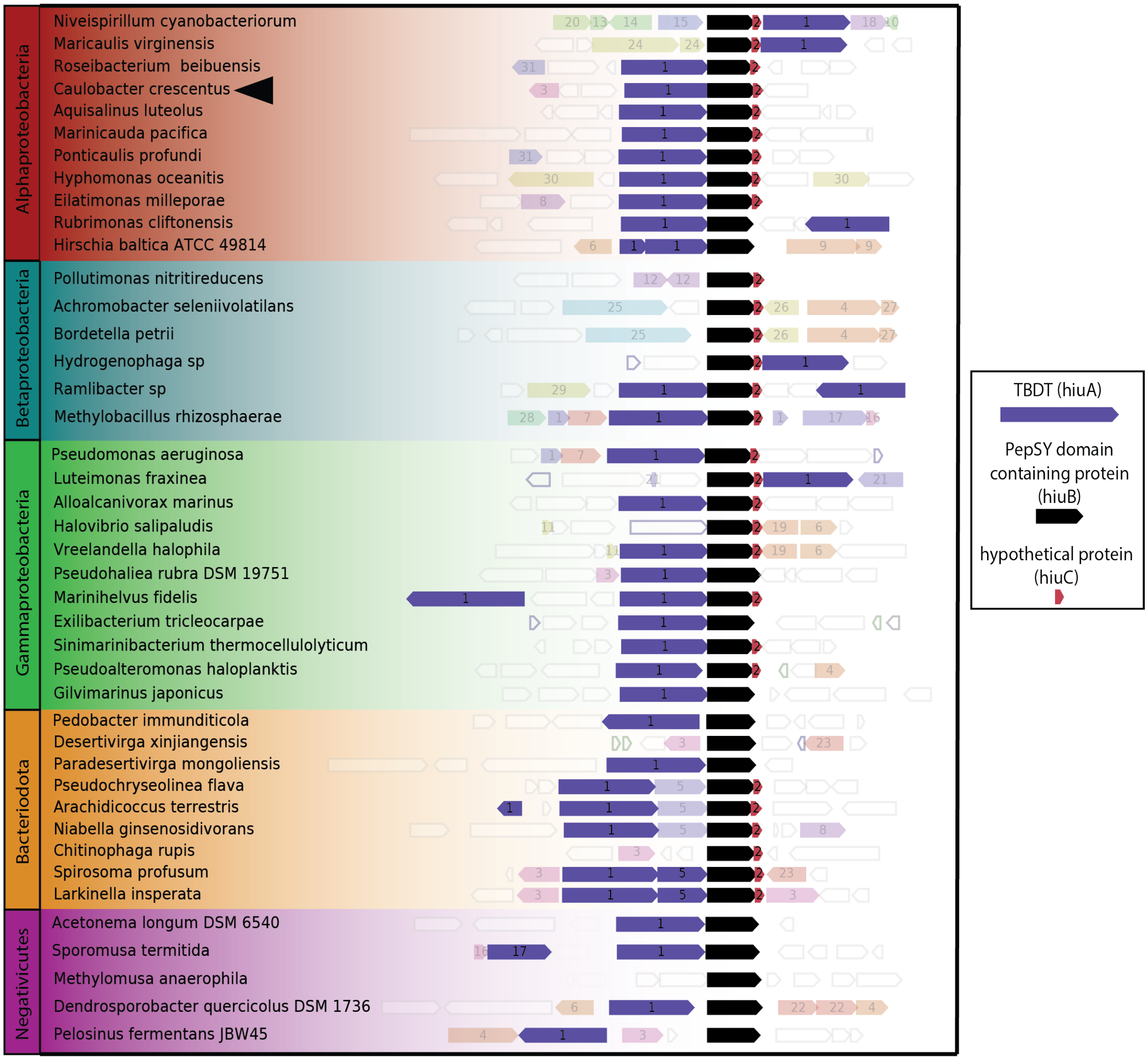

Figure S2. Conserved hiuABC-like operons are found across diverse bacterial lineages. Gene neighborhood analysis centered on *hiuB* reveals conserved synteny of *hiuA*, *hiuB*, and *hiuC* orthologs across multiple bacterial classes. Representative neighborhoods were selected based on the highest-scoring BLAST hits from members of the Proteobacteria, Bacteroidota, and Bacillota. Gene neighborhoods were visualized using webFLAGs (45). The *Caulobacter crescentus* locus is indicated by a black arrow. Orthologs of *hiuA*, *hiuB*, and *hiuC* are colored in purple, black, and red, respectively.

bioRxiv preprint doi: https://doi.org/10.1101/2025.09.04.674318; this version posted September 4, 2025. The copyright holder for this preprint (which was not certified by peer review) is the author/funder, who has granted bioRxiv a license to display the preprint in perpetuity. It is made available under aCC-BY 4.0 International license.

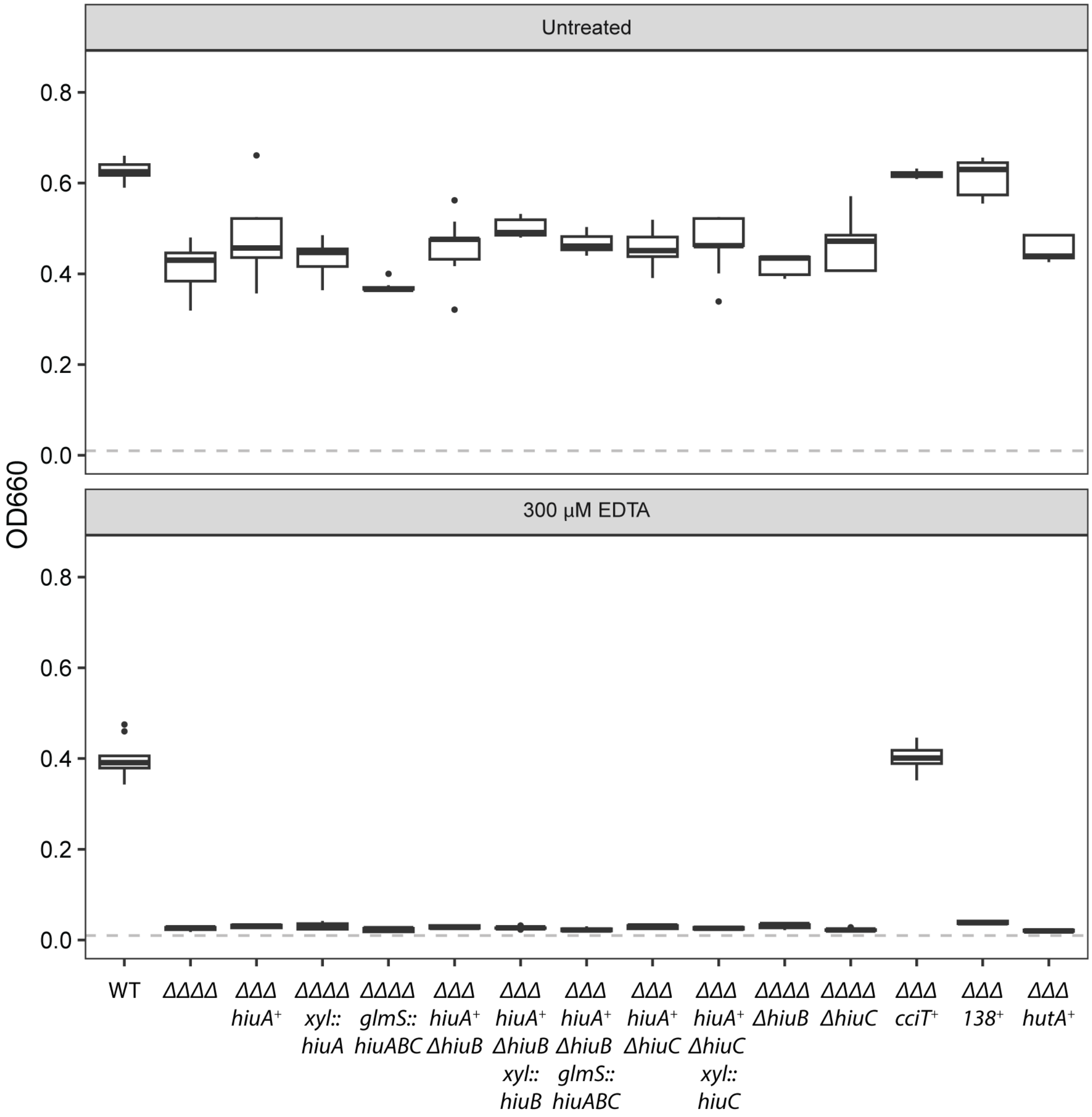

Figure S3. Growth of all *C. crescentus* strains in untreated and EDTA-treated PYE broth. Optical density at 660 nm (OD_{660}) was measured after 16 hours of growth for all *C. crescentus* strains used in this study. Full genotypes and corresponding abbreviated genotypes for all strains are included in Table S2. Cultures were inoculated at an initial OD_{660} of 0.01 (indicated by the dashed gray line) and grown in either standard PYE broth or PYE broth treated with 300 µM EDTA (B). Box plots display the median and interquartile range (25th to 75th percentiles) and are overlaid with individual data points from nine independent cultures per strain (n = 9).

bioRxiv preprint doi: https://doi.org/10.1101/2025.09.04.674318; this version posted September 4, 2025. The copyright holder for this preprint (which was not certified by peer review) is the author/funder, who has granted bioRxiv a license to display the preprint in perpetuity. It is made available under aCC-BY 4.0 International license.

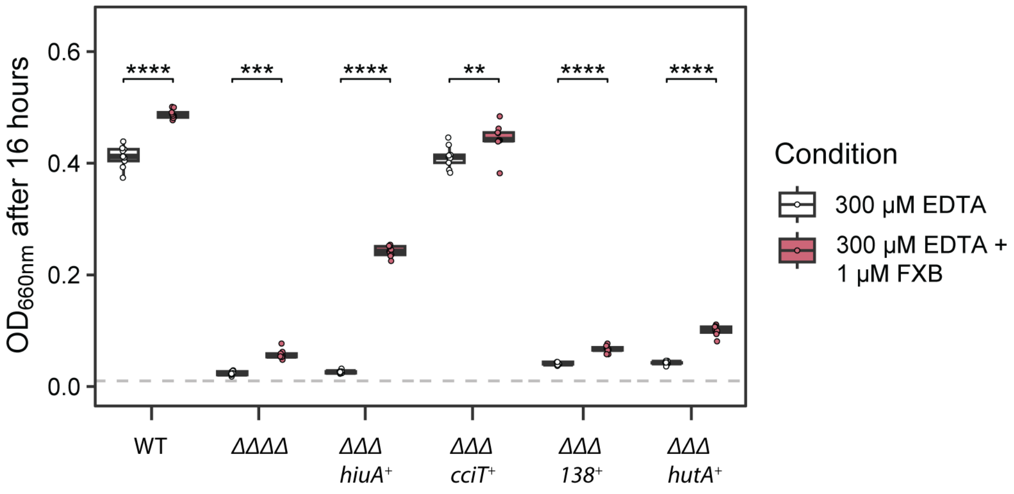

Figure S4. hiuA **confers optimal growth on Ferrioxamine B.** Optical density (OD) at 660 nm of cultures growth 16 hours in PYE broth treated with 300 µM EDTA and supplemented with 1 µM Ferrioxamine B (FXB). Strains include wild-type (WT), a strain lacking all four Fur-regulated TBDTs *(ΔΔΔΔ)*, and strains encoding only one of each of the four Fur-regulated TBDTs (*cciT, CCNA_00138, hutA, or hiuA*) and lacking the other three (*ΔΔΔ*) Fur-regulated TBDTs. Strains were inoculated at 0.01 OD represented by the dashed gray line. Box plots reflecting the median and the 25th and 75th percentiles are overlaid with the individual data points for each independent culture (n = 9). Comparison of the OD of a strain between conditions was done through an unpaired t-test (Wilcoxon rank-sum test for *cciT*+). Statistical significance is indicated as follows: **P < 0.01, ***P < 0.001, ****P < 0.0001.

bioRxiv preprint doi: https://doi.org/10.1101/2025.09.04.674318; this version posted September 4, 2025. The copyright holder for this preprint (which was not certified by peer review) is the author/funder, who has granted bioRxiv a license to display the preprint in perpetuity. It is made available under aCC-BY 4.0 International license.

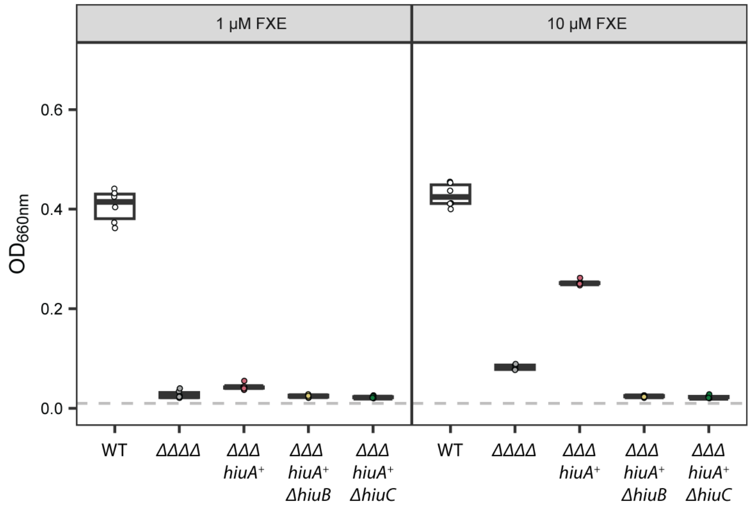

Figure S5. The *hiuABC* operon supports growth on Ferrioxamine E. Optical density (OD_{660}) of cultures grown in PYE with 300 μM EDTA and 1 μM Ferrioxamine E (FXE) or 10 μM FXE broth for 16 hours. Strains are wild-type (WT), the ΔΔΔΔ strain (lacking all four fur-regulated TonB-dependent transporters, including *hiuA*), *hiuA* $^{+}$*ΔΔΔ* (encoding only *hiuA*), strains lacking either *hiuB,* or *hiuC* in the *hiuA* $^{+}$*ΔΔΔ* background. Strains were inoculated at 0.01 OD660 represented by the dashed gray line. Box plots reflecting the median and the 25th and 75th percentiles are overlaid with the individual data points for each independent culture (n =9).
